# Fluorescence activated enrichment of CD146+ cells during expansion of human bone-marrow derived mesenchymal stromal cells augments proliferation and GAG/DNA content in chondrogenic media

**DOI:** 10.1186/1471-2474-15-322

**Published:** 2014-09-27

**Authors:** Sebastien Hagmann, Sebastian Frank, Tobias Gotterbarm, Thomas Dreher, Volker Eckstein, Babak Moradi

**Affiliations:** Department of Orthopedic and Trauma Surgery, University Hospital Heidelberg, Schlierbacher Landstrasse 200a, 69118 Heidelberg, Germany; Department of Medicine V, University Hospital Heidelberg, Heidelberg, Germany; VA Boston Healthcare System, Brigham and Women’s Hospital, Harvard Medical School, Boston, MA USA

**Keywords:** Mesenchymal stromal cells, MSC surface markers, CD146, Sorting, Osteogenic differentiation, Chondrogenic differentiation

## Abstract

**Background:**

While numerous subpopulations of BM-MSCs have been identified, the relevance of these findings regarding the functional properties remains mostly unclear. With regards to attempts of enhancing differentiation results by preselecting certain MSC subtypes, we have evaluated the efficiency of CD146 purification during expansion, and evaluated whether these measures enhanced MSC differentiation results.

**Methods:**

Human MSCs were derived from bone marrow of six donors and cultured in two different culture media. After P1, MSCs were purified by either magnetic or fluorescence sorting for CD146, with unsorted cells as controls. Growth characteristics and typical MSC surface markers were assessed from P0 to P3. After P3, chondrogenic, osteogenic and adipogenic differentiation potential were assessed.

**Results:**

Despite a high variability of CD146 expression among the donors, fluorescence sorting significantly increased the number of CD146+ cells compared to control MSCs, while magnetic sorting led to a lesser enrichment. Osteogenic and adipogenic differentiation potential was not affected by the sorting process. However, FACS-sorted cells showed significantly increased GAG/DNA content after chondrogenic differentiation compared to control MSCs.

**Conclusion:**

FACS sorting of CD146+ cells was more efficient than magnetic sorting. The underlying mechanism of increased GAG/DNA content after enrichment during expansion remains unclear, but may be linked to increased proliferation rates in these cells.

**Electronic supplementary material:**

The online version of this article (doi:10.1186/1471-2474-15-322) contains supplementary material, which is available to authorized users.

## Background

Mesenchymal stromal or stem cells (MSCs) are multipotent cells that have been isolated from various tissues, such as bone-marrow [1, 2], adipose tissue [3, 4], cord blood and tissue [2, 5] and peripheral blood [6, 7]. Their multilineage potential has led to an accelerating research in these cells in orthopaedics [8–10], cardiology [11, 12], hematology [13, 14] and neurology [15, 16], to name only a few. Additionally, MSCs were shown to possess important immunoregulatory properties [17–19], which has become another therapeutic approach.

Although initially understood as a distinct entity, more and more studies have revealed that MSCs must be understood as a heterogeneous population with multiple different subpopulations [20–25]. In the attempt to further characterize MSCs, numerous surface markers have been identified. Some of them have been included in minimal criteria for MSCs defined by the International Society for Cellular Therapy [26]. However, there have been numerous reports on new surface markers that are also applicable for characterizing MSCs [20, 27–30]. One of these novel markers described on MSCs is CD146, or melanoma cell adhesion molecule (MCAM, MUC18). It is not only recognized as a marker for endothelial progenitor cells and perivascular stem cells [31, 32], but has been shown to be a marker for MSCs as well [1, 33]. CD146 expression seems to vary in MSCs derived from different tissues [34], and was reported to distinguish MSCs from fibroblasts [35]. Previous studies of our group and other groups have also detected that CD146 expression is dependent on the composition of MSC culture media and is downregulated by FGF-2 administration during expansion [36, 37]. An ultimate goal that drives the research on MSC subtypes is to optimize the aspired tissues, thus making tissue engineering in any of these fields more effective. The isolation of MSC subtypes seems a promising technique in order to utilize specific MSC properties [38]. Two principal methods seem appropriate in this regard: isolation by magnetic or fluorescence labelling. Several studies have described methods to isolate the CD146+ fraction from endometrium [39] or the peridontal ligaments [40]. CD146+ MSCs have also been isolated from bone marrow by CD34 negative sorting [1].

However, up to date, it has not yet been sufficiently demonstrated whether the CD146+ MSCs exert distinct differentiation properties. Additionally, it is still unclear which isolation method (fluorescence or magnetic) is more favourable to accumulate CD146+ MSC subsets from bone marrow.

We therefore investigated the impact of a CD146+ cell purification during expansion of human bone-marrow derived MSCs. It was further evaluated if chondrogenic, osteogenic and adipogenic differentiation of the MSCs were affected. Furthermore, we employed two different expansion media, one with a simple and one with a more elaborate formula, to evaluate if the methods were suitable for different culture conditions.

## Methods

### Bone marrow donors

A total of n = 6 donors (mean age 62.2 ± 16.4 years, 3 female, 3 male donors) were included in the study. Bone marrow aspiration was performed from the femur during total hip arthroplasty (for end-stage osteoarthritis) or from the iliac crest during autologous bone grafting (for enhancing healing in an osteotomy). All donors approved written informed consent prior to bone marrow donation. The study protocol was approved by the ethics committee of the University of Heidelberg and was conducted according to the latest version of the Helsinki Declaration.

### Isolation of human bone marrow-derived MSCs

Bone marrow was collected into syringes containing 5000 I.E. heparine (ratiopharm, Ulm, Germany) and diluted in “Ringer” isotonic saline solution (Braun, Melsungen, Germany). After washing with PBS (Invitrogen, Karlsruhe, Germany), bone marrow mononuclear cells (BM-MNCs) were collected from the interphase created by Ficoll paque plus gradient centrifugation (GE Healthcare, Uppsala, Sweden) and washed in PBS. Washing was repeated twice; afterwards the cells were resuspended in PBS and counted in triplicates in a Neubauer chamber (Brand, Wertheim, Germany) after staining with Tuerk solution (Sigma-Aldrich, Schnelldorf, Germany). BM-MNCs of each donor were resuspended in two different culture media (shown below) at a density of 1.25×10^5^ cells /cm^2^ in T75 cell culture flasks (Greiner Bio One, Frickenhausen, Germany). The cells were cultured in a humidified thermostat at 37°C and 6% CO2. After 24 hours, medium replacement was performed and only adherent cells remained in cell culture. Cells were inspected by polarization microscopy daily. At 80% confluence, cells were detached with trypsine/EDTA solution (Biochrom, Berlin, Germany) after washing with PBS. Whole medium was added, and the cells were counted as described above. The cells were then washed in whole medium and resuspended at a density of 5×10^4^ cells/cm^2^ in the respective media. These procedures were repeated until the end of passage 3.

The cells of each donor were cultured in a) Dulbecco’s modified Eagle’s medium low glucose (DMEM-LG, Invitrogen, Karlsruhe, Germany) with 20% fetal calf serum (FCS, Invitrogen, Karlsruhe, Germany) and 1% penicilline/streptomycine (Invitrogen, Karlsruhe, Germany) and b) a variation of Embryonal Stem Cell expansion medium (ES), consisting of DMEM-high glucose (DMEM-HG, Invitrogen, Karlsruhe, Germany) 12.5% FCS, 2 mM L-glutamin, 50 mM b-mercaptoethanol, 1% nonessential amino acids 100, 1% penicilline/streptomycine (all Invitrogen, Karlsruhe, Germany), and 4 ng/ml basic fibroblast growth factor (bFGF/FGF-2, Acris, Herford, Germany). As a marker for proliferation, a growth index per day (GID) was calculated through the formula: GID = (number of cells at beginning of passage/number of cells at end of passage)/days in passage. For the sorted groups, a relative GID was then calculated by a quotient of the respective GID and the GID of untreated control cells to allow comparability among the donors.

As a positive control for CD146 positive cells, HeLa cells were cultured in medium condition a) and b) for 63 days. Fluorescence cytometry for CD146 (see below) was conducted weekly throughout this time.

### CD146 isolation of BM-MSCs

In addition to the culture conditions detailed above, two different isolation techniques for BM-MSCs during their expansion. An initial experiment showed that the purification in both media was more effective in P1 than in P0 (data not shown), which is why the separation process was performed in P1. After detaching, counting and washing the cells with whole medium, cells were separated into three parts per medium. For the first group (control group), MSCs were resuspended as described above. The second group underwent magnetic sorting for CD146+ cells. MSCs were therefore washed with PBS and incubated with 20 μl of FcR blocking reagent human (Miltenyi Biotec, Bergisch Gladbach, Germany) per 10^7^ cells for 5 min. Afterwards, MSCs were washed in auto MACS running buffer (Miltenyi Biotec, Bergisch Gladbach, Germany), and 20 μl of CD146 microbeads (Miltenyi Biotec, Bergisch Gladbach, Germany) per 10^7^ cells were added. After an incubation of 15 min, cells were washed and resuspended in the auto MACS buffer, and magnetic isolation was performed according to manufacturers’ protocols using LS columns and a MidiMACS™ separator (Miltenyi Biotec, Bergisch Gladbach, Germany). Afterwards, MSCs were washed in MACS buffer. After this separation step, cells were analysed by flow cytometry (see below) and further cultured in the same manner as the control group.

The third group underwent fluorescence cell sorting. MSCs were therefore washed with PBS and incubated with 20 μl of FcR blocking reagent per 10^7^ cells for 5 min. Afterwards, MSCs were washed and resuspended in auto MACS buffer. Cells were then stained with 10 μl/10^6^ cells of a mouse anti-human CD146 PE antibody (BD Biosciences, Heidelberg, Germany) for 30 min at 4°C in the dark. MSCs were then washed and fluorescence cell sorting was performed using a FACS Aria II sorter (BD Biosciences, Heidelberg, Germany) and the FACSDiva software (Version 6.1.3., BD Biosciences, Heidelberg, Germany). Following this separation step, MSCs were analysed by flow cytometry (see below) and further cultured in the same manner as the control group.

### Flow cytometry

**Figure 1:**
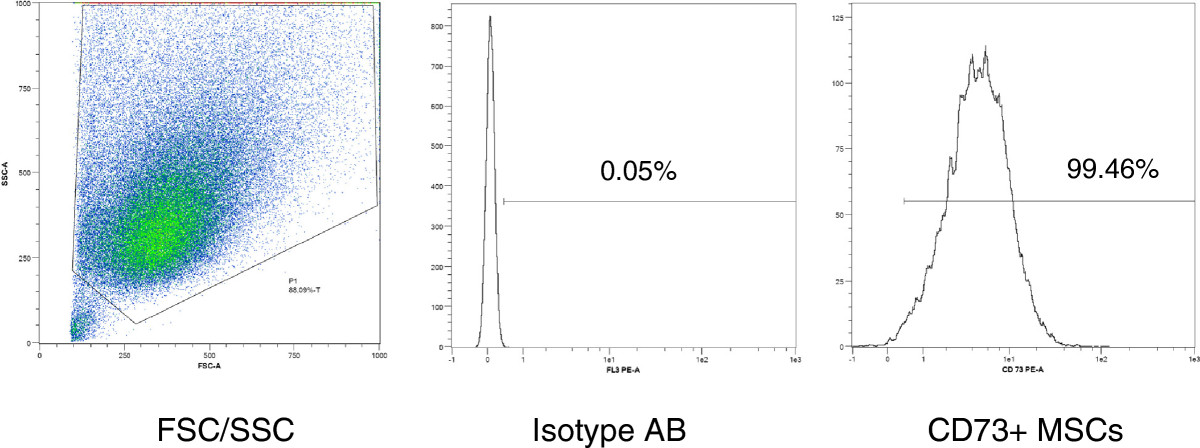
**Gating strategy for flow cytometry analysis of MSCs** ***.*** FSC/SSC: forward scatter/sideward scatter. Isotype AB: isotype control antibody. The bar defines the border of 99.5% of the isotype control fluorescence signal. In this example, 99.46% of the MSCs express CD73.

Confirmation of purification for CD146 was performed by flow cytometry in all groups after separation. For cell saving reasons, flow cytometry was performed for the entirety of the antibodies listed below in P0, P2 and P3, and in the control MSCs after P1. MSCs were suspended in PBS with 0.5% FCS and 2 mM EDTA and labelled with the following mouse anti-human antibodies: CD14 FITC, CD34 PE, CD45 APC-Cy™7, CD90 FITC, CD73 PE, CD105 PerCP-Cy™5 and CD146 PE (all BD Biosciences, Heidelberg, Germany). Cell viability was assessed using a 7AAD Viability Staining Solution (eBioscience, Frankfurt, Germany). Flow cytometry was conducted with a MACS Quant™ analyser and the MACS Quantify 2.1 software (Miltenyi Biotec, Bergisch Gladbach, Germany). Isotype matched antibodies were employed for background fluorescence detection. Positive fluorescence was defined as any event occurring above a gate defined by containing 99.5% of the events measured for background fluorescence in a histogram plot. The gating strategy is demonstrated in Figure 1.

### Chondrogenic differentiation

MSCs were detached from the culture flasks with trypsine/EDTA solution as described above, washed and resuspended in whole medium. 5×10^5^ cells were then centrifuged at 3000/min for 5 min for high density pellet culture and then cultured in chondrogenic medium, which consisted of 286 ml DMEM HG (Invitrogen, Karlsruhe, Germany), 5 μg/ml transferrin, 5 ng/ml sodium selenite, 1 mM Sodium pyruvate (all Sigma-Aldrich, Schnelldorf, Germany), 1,25 mg/ml BSA, 100 units/ml P/S (both Invitrogen, Karlsruhe, Germany), supplemented by 0,1 μM dexamethasone, 5 μl ascorbic acid/5 ml (both Sigma-Aldrich, Schnelldorf, Germany), 10 ng/ml TGF-β (Acris, Herford, Germany) and 5 μg/ml insuline glargin (Sanofi Aventis, Frankfurt, Germany). Chondrogenic medium was changed three times a week, and MSCs were incubated for 42 days. N = 2-5 pellets per group were analysed, and intraindividual means were calculated. For quantitative analysis of GAG deposition, the pellets were digested with pepsin solution overnight and then stained with 1,9-dimethyl-methylene blue (dye content 80%, Sigma-Aldrich, Schnelldorf, Germany). Absorption was measured at 530 nm for the pellets and a chondroitin 4-sulfate standard (Sigma-Aldrich, Schnelldorf, Germany). DNA content in the pellets was analyzed with a Quant iT ds Pico Green DNA Assay Kit (Invitrogen, Karlsruhe, Germany) according to manufacturers’ protocols, and GAG/DNA content was calculated.

### Osteogenic differentiation

MSCs were harvested with trypsine/EDTA solution as described above, washed and resuspended in whole medium. 35,000 MSCs per well were seeded in 24 well plates (Nunclon Surface, Sigma Aldrich, Schnelldorf, Germany) containing 0.5 ml of osteogenic induction medium. Four assays per time point were conducted. Osteogenesis was induced with a medium consisting of DMEM HG, 10% FCS, 1% penicilline/streptomycine (all Invitrogen, Karlsruhe, Germany), 0.1 mM dexamethasone, 0.17 mM ascorbic acid 2-phosphate, and 10 mM β-glycerophosphate (all Sigma-Aldrich, Schnelldorf, Germany). Osteogenesis was induced for 21 days, quantified by an alkaline phosphatase assay and alizarin red staining at d3, d7, d14 and d21. MSCs therefore were lysated in 0.5 ml 1% Triton X-100 (Sigma-Aldrich, Schnelldorf, Germany), and the lysate was incubated 1:1 with 1 mg/ml p-nitrophenylphosphate in ALP-buffer (0.1 M glycine, 1 mM MgCl2, 1 mM ZnCl2, pH 10.4). Substrate turnover was measured in an MRX ELISA reader (Dynatech Laboratories, Stuttgart, Germany) at 405/490 nm, and results were calculated with an ALP standard. ALP levels were standardized to protein content, which was analyzed using a Micro BCA Protein Assay Kit (Pierce, Rockford, USA) according to manufacturers’ instructions. Calcium deposition was quantified by 0.5% Alizarin Red S staining (Sigma-Aldrich, Schnelldorf, Germany) at 570 nm, calculated according to an Alizarin standard and then standardized to whole protein content as described above.

### Adipogenic differentiation

MSCs were seeded with 35,000 cells per well in 24 well plates (Nunclon Surface, Sigma Aldrich, Schnelldorf, Germany) in adipogenic induction medium and cultured for 14 days (n = 2-4 assays per donor). Adipogenic differentiation medium consisted of DMEM HG (Invitrogen, Karlsruhe, Germany), 10% FCS (Invitrogen, Karlsruhe, Germany), 1 mM dexamethasone, 0.2 mM indomethacine, 0.5 mM isobutyl methylxanthine (all Sigma-Aldrich, Schnelldorf, Germany) 0.01 mg/ml insulin glargin (Sanofi-Aventis, Frankfurt, Germany) and 1% penicilline/streptomycine (Biochrom, Berlin, Germany).

Adipogenic differentiation was assessed by fixation with 4% paraformaldehyde and staining with 0.3% Oil Red O solution (Chroma, Münster, Germany). Evaluation of adipogenic differentiation was conducted by a qualitative microscopic assessment of lipid vacuole formation.

### Statistical analysis

Statistical analysis was performed with the SPSS computer software (SPSS Inc., released 2009, PASW Statistics for Windows, Version 18.0. Chicago). QQ-plots, box plots, a ratio analysis and Kolmogorov-Smirnov (with Lilliefors significance correction) as well as Shapiro-Wilk tests were performed to evaluate normal distribution of the data. For parametric data, two-tailed paired t-tests were performed for comparisons between two different media conditions, while analyses of variance (ANOVA) followed by Bonferroni correction were performed for comparisons of more than two groups (population doubling per passage, passage time, ALP content and calcium deposition). Non-parametrical data was analysed by Wilcoxon tests for the comparison of two and Friedman tests for the comparison of more than two groups (surface marker expression, relative GAG/DNA content). Differences were considered statistically significant for p-values smaller 0.05. Results are shown as means ± standard deviation.

## Results

### Proliferation rates

**Figure 2:**
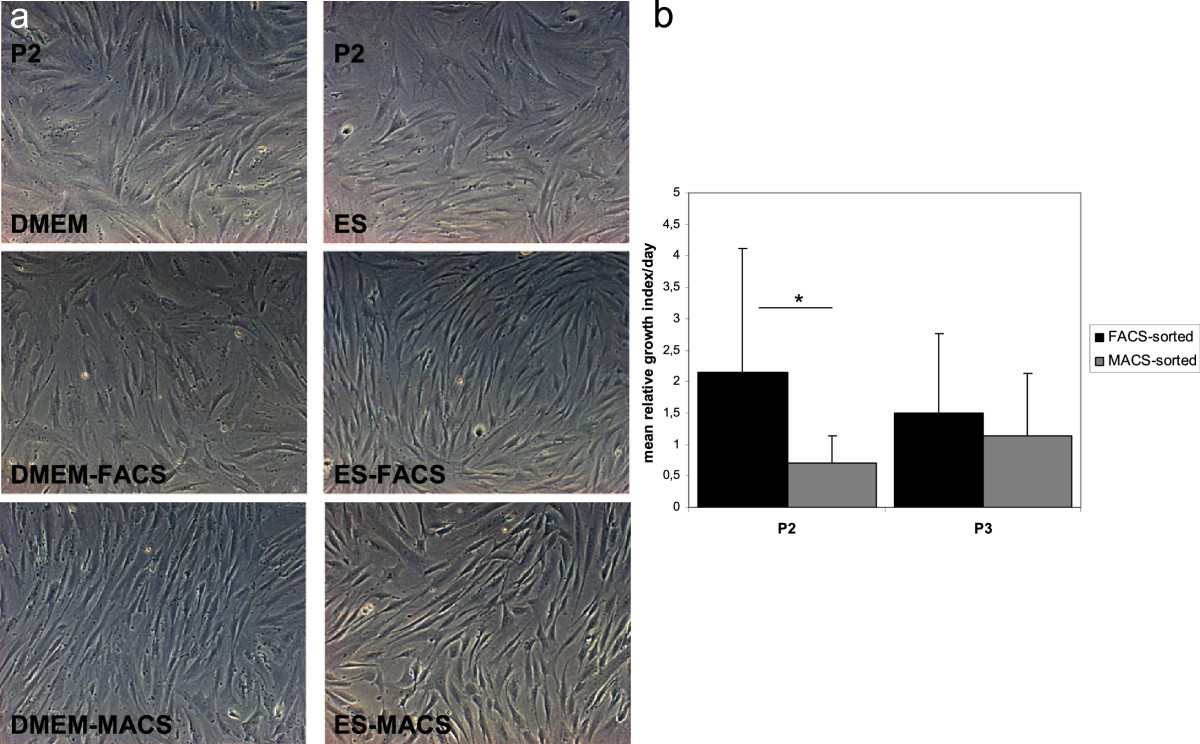
**Morphological aspects of MSCs and proliferation rates. a)** MSCs sorted for CD146 by fluorescence cytometry (DMEM-FACS and ES-FACS) or magnetically (DMEM-MACS and ES-MACS) showed no morphological differences to the control MSCs cultured in DMEM-LG or ES medium. Light microscopy, magnification x100. **b)** Results displayed are the means and SD of the growth index per day divided by the growth index of control cells for each respective donor*.* *p < 0.05.

No morphological differences were observed between sorted and unsorted cells and between the two media conditions (Figure 2). No significant differences concerning proliferation were observed between the two media conditions. The mean relative growth index per day was higher in FACS-separated cells and lower in MACS-separated cells in P2, resulting in a significantly higher proliferation of FACS vs. MACS-sorted cells (Figure 2, p = 0.022). MACS-sorted cells closed up to FACS-sorted MSCs in P3 (Figure 2, comparison MACS/FACS: p = 0.388).

### MSC surface marker expression

**Figure 3:**
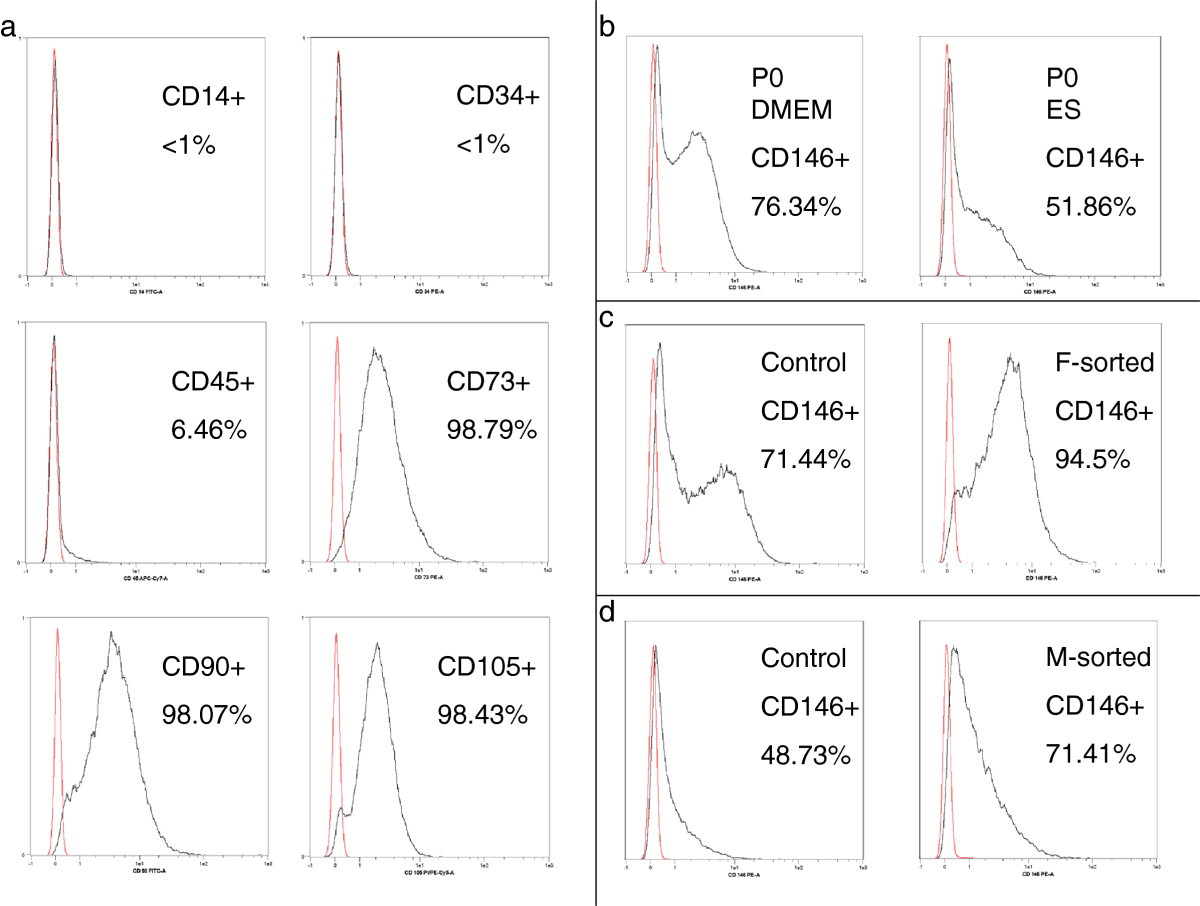
**Surface marker distribution on MSCs. a)** MSCs were clearly negative for CD14 and CD34 while CD45 was expressed on some of the cells. MSCs were positive for CD73, CD90 and CD105. The histograms displayed are P2 control MSCs cultured in DMEM-LG. All histograms have been adjusted for height. **b)** Representative histograms from P0 cells in DMEM and ES medium. CD146 expression was significantly lower in ES than in DMEM in P0. **c)** Exemplary histograms of P2 control vs. FACS-sorted MSCs. **d)** Exemplary histograms of P2 control vs. MACS-sorted MSCs. There was an important variation of CD146 expression between patients, as demonstrated by the P2 samples from c and d. Magnetic sorting resulted in a lower purification than fluorescence sorting, while higher CD146 expression in naïve MSCs resulted in a higher purification result.

Representative histograms for the surface markers are shown in Figure 3. Except for CD34, which was consistently expressed on less than 1% of the MSCs in all passages and in all donors, an important variation of the other surface markers could be observed. The P0 populations showed a more heterogeneous surface marker profile than P1, P2 and P3 populations regarding CD73, CD90, CD14 and CD45 in both media (Table 1). No differences between the groups were observed for 7-AAD positive cells; however both media and all groups showed a mild increase in 7-AAD positive cells from under 1% to 4% from P0 to P3 (data not shown). CD45 expression was markedly higher than the other negative markers (Table 1, Figure 3), with no significant differences between the media.

**Table 1:** **Surface marker expression in P0, P1, P2 and P3 in DMEM and ES media (see text for media composition)**

DMEM	P0	P1	P2	P3	p value
					P0/P1, P0/P2, P0/P3
CD73	85.41 ± 10.96	97.34 ± 1.96	97.83 ± 3.62	99.3 ± 0.62	0.068, **0.028**, 0.109
CD90	89.78 ± 6.52	98.39 ± 0.44	98.89 ± 0.69	98.18 ± 1.62	0.068, **0.028, 0.043**
CD105	89.02 ± 17.18	95.45 ± 4.34	97.72 ± 1.53	98.33 ± 0.57	0.715, 0.249, 0.686
CD146	72.98 ± 9.21	78.62 ± 10.42	74.3 ± 17.31	72.64 ± 20	0.068, 0.917, 0.893
CD14	9.66 ± 7.61	1.21 ± 0.7	<1	<1	0.068, **0.028, 0.028**
CD34	<1	<1	<1	<1	N/A
CD45	12.69 ± 8.44	5.53 ± 1.59	6.03 ± 2.34	6.16 ± 4.1	0.144, 0.249, 0.249
**ES**	P0	P1	P2	P3	p value
					P0/P1, P0/P2, P0/P3
CD73	85.75 ± 11.27	99.045 ± 0.8	99.41 ± 0.6	99.26 ± 0.29	0.068, **0.043**, 0.109
CD90	78.72 ± 9.04	94.1 ± 2.37	94.98 ± 4.18	97.3 ± 2.54	0.109, **0.075**, 0.109
CD105	89.06 ± 8.77	96.97 ± 2.89	96.8 ± 2.63	96.19 ± 4.84	0.068, 0.116, 0.285
CD146	56.07 ± 7.69	60.54 ± 7.59	55.35 ± 11.91	45.19 ± 15.38	0.068, 0.753, 0.465
CD14	9.55 ± 6.93	2.13 ± 2.11	<1	<1	0.068, **0.028**, 0.109
CD34	<1	<1	<1	<1	N/A
CD45	12.14 ± 8.77	3.5 ± 2.04	2.46 ± 1.54	5.19 ± 3.8	0.109, **0.028**, 0.273

Results are displayed as mean positive cells (%) ± SD.

Bold p-values represent results below p=0.05.

No significant differences regarding surface marker distribution could be detected between the two media except for CD90, which showed a higher expression in P0 in cells cultured in DMEM compared to ES medium (89.78 ± 6.52% vs. 78.72.02 ± 9.04%, p = 0.028).

**Figure 4:**
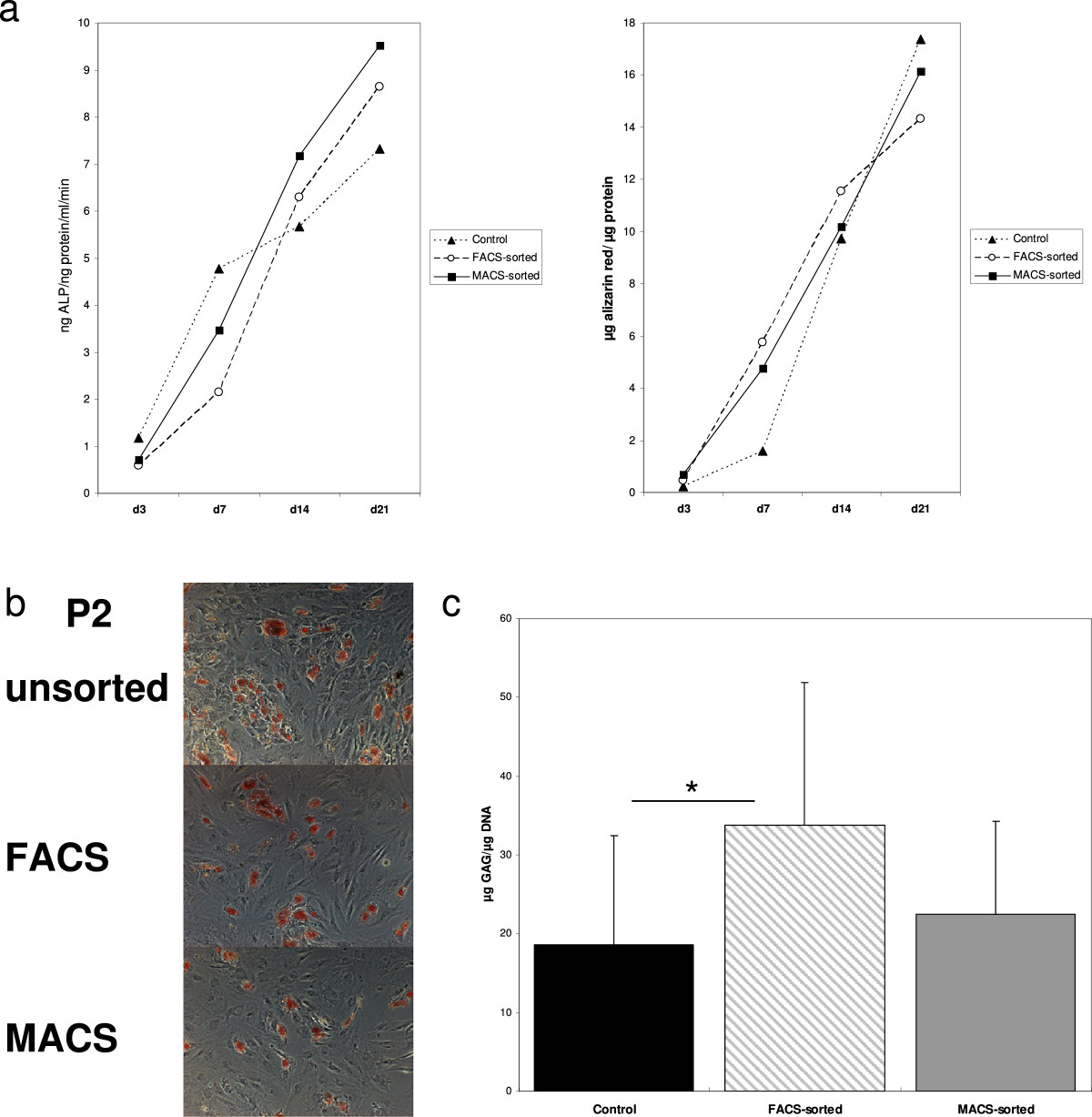
**Osteogenic, adipogenic and chondrogenic differentiation results. a)** Alizarin red contents per protein at days 3, 7, 14 and 21 are displayed in the right diagram, ALP contents per protein per ml and min at days 3, 7, 14 and 21 are displayed in the left diagram. No significant differences between fluorescence or magnetic sorting and the control MSCs could be detected. **b)** No differences regarding adipogenic differentiation of MSCs cultured in DMEM or ES medium and sorted for CD146 by fluorescence cytometry (DMEM-FACS and ES-FACS) or magnetically (DMEM-MACS and ES-MACS) could be detected. Light microscopy, magnification x100. **c)** Mean GAG/DNA content of control MSCs, FACS and MACS sorted cells after 21 days of chondrogenic differentiation is displayed. *p < 0.05.

The greatest heterogeneity of the MSC preparations was observed for CD146, which was highly donor-dependent (expression ranging from 48.69% to 89.43% in P0, Figure 4). While no significant differences between the passages could be detected, CD146 expression was higher when MSCs were cultured in DMEM compared to ES medium in P0 and P2 (Table 1, Figure 3, P0 and P2: p = 0.028).

The HeLa cell cultures showed equally high CD146 expression in both culture conditions over 63 days (means: 99.39 ± 0.74% in condition a, 99.04 ± 0.98% in condition b, 97; p = 0.373).

### Magnetic vs. fluorescence separation

Representative sorting results for both methods are shown in Figure 3. Due to the important heterogeneity of CD146 expression among the donors, individual purification results were calculated for each donor. The mean overall purification effect of fluorescence sorting for CD146 in all donors was factor 1.8 (±0.62) compared to the pre-sorting results (p = 0.002). Purification ranged from factor 1.17 in a donor with high CD146 expression to factor 3.27 in the donor with the lowest CD146 expression.

MACS-sorting did only slightly enhance the number of CD146 expressing cells, with a mean overall purification of factor 1.09 (±0.35) (p = 0.646 compared to the control group).

Neither magnetic nor fluorescence cell sorting affected the distribution of any of the other surface markers, which were consistently high for the positive markers CD73, CD90 and CD105 and consistently low for the negative markers CD14 and CD34, while all groups showed a CD45 expression of under 10%, comparable to the control cells (data not shown).

### Differentiation results

All groups were successfully differentiated into adipogenic, chondrogenic and osteogenic lineage. There was no apparent difference in adipogenic differentiation in both media conditions and depending on whether and which CD146+ separation was applied, as reflected by optical density analysis and qualitative assessment of lipid vacuole formation (Figure 4). All groups showed adequate ALP increase and calcium deposition over time. No significant differences regarding osteogenic differentiation were observed (Figure 4).There was an important variation in chondrogenesis related to the donors. When compared to the control MSCs, FACS-sorted cells showed higher GAG/DNA content (Figure 4, p = 0.023). No significant differences concerning GAG/DNA content could be observed when comparing MACS-sorted cells to the control MSCs (Figure 4, p = 0.117). Neither in MACS-, nor in FACS-sorted cells, GAG/DNA content correlated with the expression of any of the surface markers MSCs were analyzed for, including CD146 (Pearson Correlation for P3 -0.290, p = 0.096).

## Discussion

MSCs are promising for applications in medicine both for their regenerative and immunoregulatory properties. While the use of these cells in human subjects increases, there is an ongoing controversy about whether the phenotypic heterogeneity of these cells may be a benefit or a disadvantage for these capacities. In order to better understand the nature of MSC subpopulations, attempts have been made to compare homogenous MSC preparations to crude MSCs. The aim of our study was to evaluate two different methods to purify bone-marrow derived MSCs for CD146, a marker that has been associated with endothelial cells, but recently with MSCs as well [41, 42]. Fluorescence sorting provided a significant increase in CD146 expression, an increase in proliferation and chondrogenic differentiation, while osteogenic and adipogenic differentiation remained unchanged.

Our experiments focussed on a comparison of two different isolation techniques for CD146. In contrast to Sorrentino et al. [1], who derived a purified CD146+ population by sorting out CD34+ cells from an enriched BM-MSC population, we chose to directly sort the cells by positive selection for CD146 to determine whether these procedures affected the functional characteristics of the MSCs. While we observed an important donor-related variation of CD146 expression in both media, a major difference between our experiments and the above study was also that the initial P0 CD146 expression level on BM-MSCs was relatively high, which is in accordance with our own findings from previous studies and with reports by Halfon et al. 2011 [35]. In contrary, other studies have found natural CD146 expression levels of BM-MSCs around 15% in P0 [41]. These differences may be due to different isolation and expansion techniques, but also due to different donor populations [43–45]. Further research is needed to determine why such striking differences in CD146 expression of human BM-MSCs have been reported in different studies.

Baksh et al. reported an easy feasibility of CD146 positive and negative sorting with fluorescence sorting [41], which is in accordance with our findings. In contrast, magnetic sorting led to a much lower increase in CD146 expression. In our opinion, this is an effect mainly encountered with MSCs, which are more heterogeneous than lymphocytes, where magnetic sorting is more effective. A limitation in both groups was that the natural CD146 expression before the sorting process showed a high donor-dependency, which resulted in a high purification in donors with a low CD146 expression, while the effect was less when CD146 expression was already high. The variations in surface marker distribution and growth as well as differentiation parameters are in accordance with the donor-related heterogeneity of MSCs described by other groups [43, 44]. Interestingly, CD146 expression may also be one of the distinct properties of MSCs derived from different tissues [46, 47].

In 2010, Russell et al. reported CD146 being associated with higher potency of bone-marrow derived MSCs, while all other surface markers, among them CD44, CD73 and CD271 showed no correlation with proliferation and colony-forming unit efficiency [48]. This is in accordance with our findings, where we observed a higher proliferation of MSCs sorted by fluorescence sorting. Considering the differentiation potential, our findings suggest that both methods retain the osteogenic and adipogenic differentiation potential of BM-MSCs when compared to control MSCs. As for the chondrogenic differentiation, although important donor-dependent variations in GAG/DNA content were observed, fluorescence sorting for CD146 led to an increase of GAG/DNA content compared to control MSCs. While there was no direct correlation between any of the surface markers, including CD146, and chondrogenic differentiation, the higher proliferation rates may have contributed to the observed superior chondrogenic differentiation in the FACS-sorted cells [49].

To rule out that the effects observed were caused by an increase or decrease in viable cells, we analyzed the viability by 7-AAD staining. We did not observe differences between the media; furthermore, when compared to control MSCs, no increase in 7-AAD positive cells was observed in both sorting groups, which reflects that the separation did not lead to late apoptosis or necrosis.

We also report significant differences in MSC surface marker distribution between DMEM-LG and ES medium. We chose the two media to examine the two separation techniques in a very simple expansion medium as well as in a more complex medium. Differences in surface marker distribution of CD44, MAB1470, STRO-1 and HLA-DR have been reported depending on the media conditions applied [50]; and especially FGF-2 supplementation to the expansion media has been associated with CD146 downregulation [36]. The use of FGF-2 in ES medium may therefore have accounted for the differences in surface marker distribution. However, our results indicate that these differences are not limited to this marker, but apply to important MSC markers such as CD90 as well. With regards to their increasing use in human subjects, we believe that it is an important finding that the choice of media preselects the phenotype of MSCs. Although the functional consequences of this preselection remain unclear, the fact that the mean CD90 expression for instance in ES medium was 11% lower in ES than in DMEM-LG (with an important donor-dependant variation as well) would, according to the ISCT minimal criteria for MSCs [26] imply that ES medium provides 11% less MSCs than DMEM-LG in P0. However, our data also indicate that the expression of certain markers such as CD90 and CD73 seems to be acquired over time during in vitro culture in a number of MSCs.

Our results once again reveal the heterogeneous nature of MSCs derived from bone marrow. This heterogeneity may water some of the assumed connections between phenotype and function in many of the experiments so far. Up to this day, it has not fully become clear how regenerative features of MSCs in vitro can be predicted by their phenotype, secretion profile, or other properties. Several studies have shown that the loss of multi-potency with continued passage is not associated with changes in MSC phenotype [51, 52]. While MSC phenotype can not predict the ability of in vivo bone formation [53], other studies suggest that a certain phenotype can discriminate MSCs with adipogenic and chondrogenic potential in vitro [54]. Our study demonstrates that differentiation results can be enhanced through the purification of MSC subpopulations. While there is no direct connection to the expression of CD146, and the other surface markers did not provide evidence for subpopulations within this distinct population, other surface markers may provide this information in the future.

## Conclusion

Fluorescence activated sorting for CD146 is an adequate technique to modify tissue engineering results with regards to chondrogenic differentiation without altering osteogenic and adipogenic differentiation. While working with different MSC subpopulations may clarify the so far obscure connections between phenotype and function, our findings suggest that greater efforts to improve standardized MSC culture techniques are necessary when it comes to taking these cells into the patient.

## Authors’ original submitted files for images

Below are the links to the authors’ original submitted files for images. Authors’ original file for figure 1 Authors’ original file for figure 2 Authors’ original file for figure 3 Authors’ original file for figure 4
