# Supplementary figures and images for: Fluorescence activated enrichment of CD146+ cells during expansion of human bone-marrow derived mesenchymal stromal cells augments proliferation and GAG/DNA content in chondrogenic media
Source: BMC Musculoskelet Disord. 2014 Sep 27;15:322. doi: 10.1186/1471-2474-15-322 (PMC4196082; doi:10.1186/1471-2474-15-322)

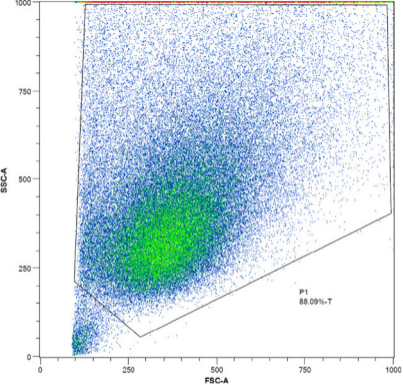

FSC/SSC

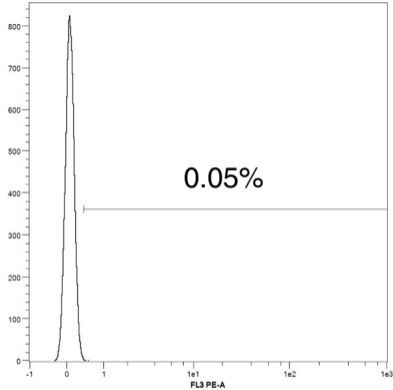

Isotype AB

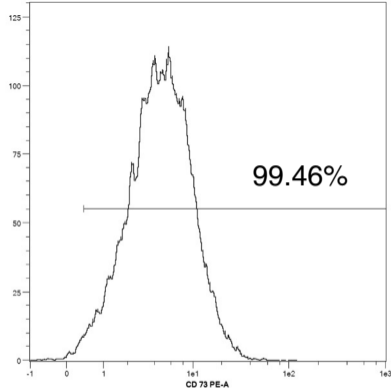

CD73+ MSCs

Supplement: Supplementary file 1 — Authors’ original file for figure 1 [file 12891_2014_2269_MOESM1_ESM.pdf]

**a****P2****DMEM**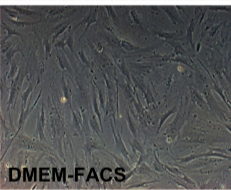**DMEM-FACS**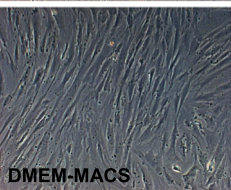**DMEM-MACS****P2****ES**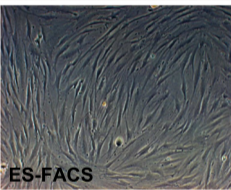**ES-FACS**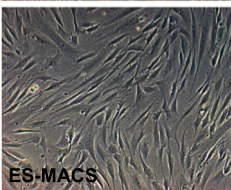**ES-MACS****b**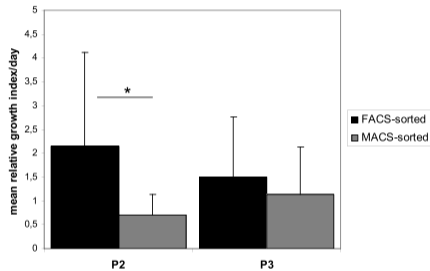

Supplement: Supplementary file 2 — Authors’ original file for figure 2 [file 12891_2014_2269_MOESM2_ESM.pdf]

**a**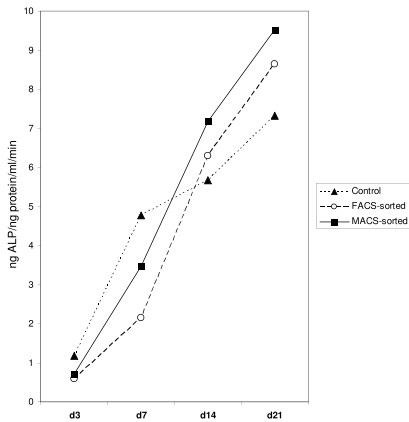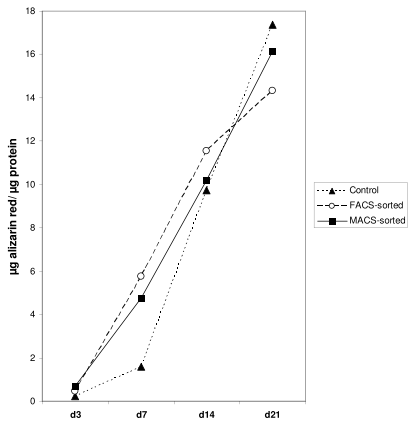**b****P2****unsorted****FACS****MACS**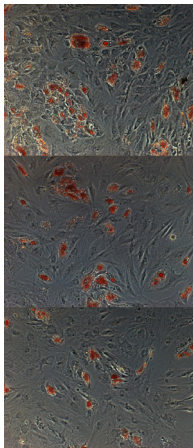**c**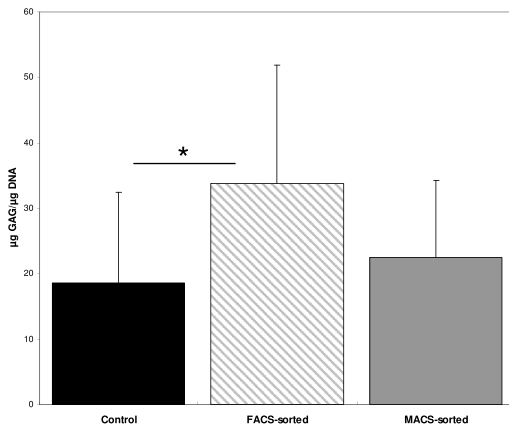

Supplement: Supplementary file 4 — Authors’ original file for figure 4 [file 12891_2014_2269_MOESM4_ESM.pdf]
