# Supplementary material for: Fluorescence activated enrichment of CD146+ cells during expansion of human bone-marrow derived mesenchymal stromal cells augments proliferation and GAG/DNA content in chondrogenic media
Source: BMC Musculoskelet Disord. 2014 Sep 27;15:322. doi: 10.1186/1471-2474-15-322 (PMC4196082; doi:10.1186/1471-2474-15-322)
Supplement: Supplementary file 3 — Authors’ original file for figure 3 [file 12891_2014_2269_MOESM3_ESM.pdf]

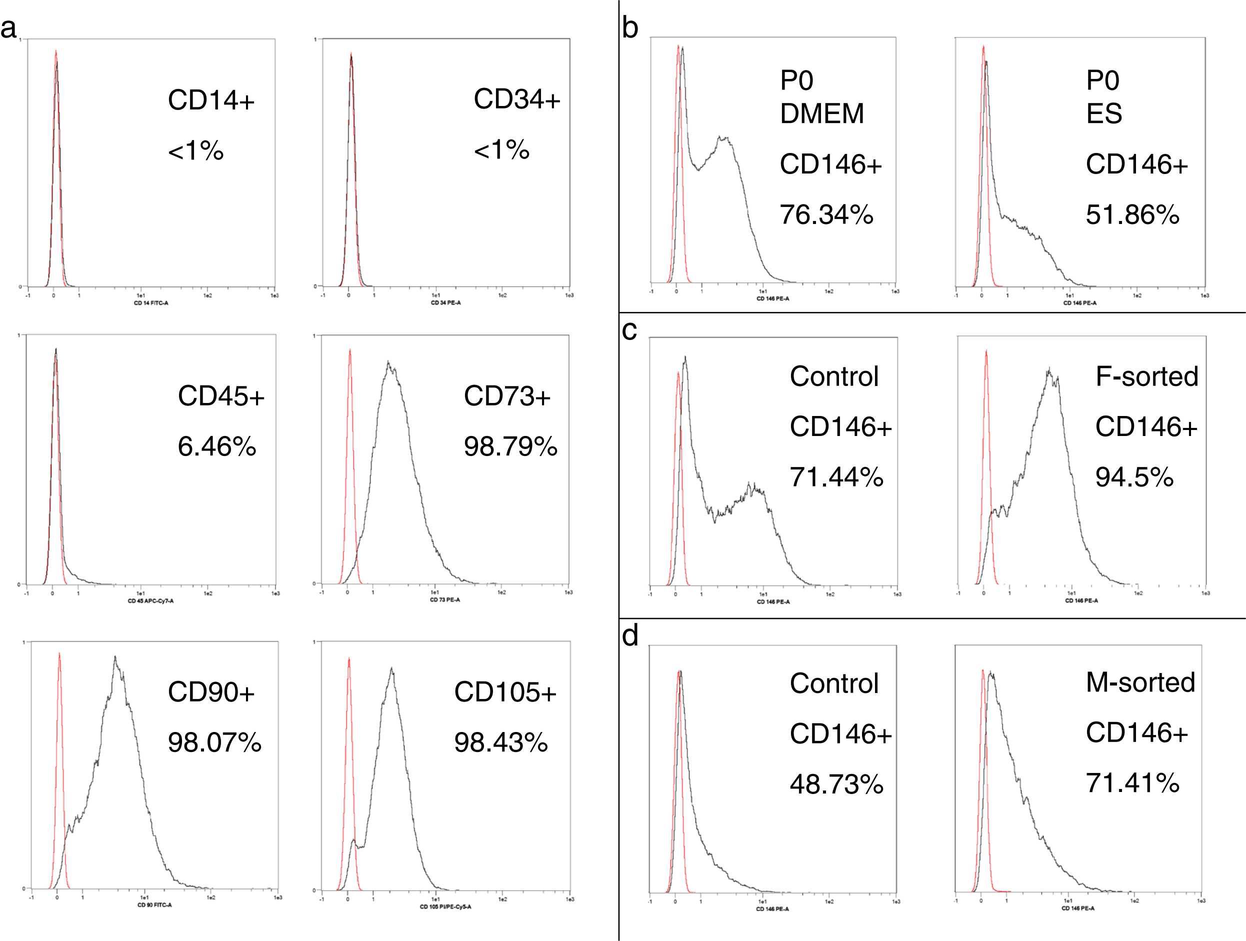
a
CD14+
<1%
CD 14 FITC-A
CD34+
<1%
CD 34 PE-A
CD45+
6.46%
CD 45 APC-Cy7-A
CD73+
98.79%
CD 73 PE-A
CD90+
98.07%
CD 90 FITC-A
CD105+
98.43%
CD 105 PI/PE-Cy5-A
b
P0
DMEM
CD146+
76.34%
P0
ES
CD146+
51.86%
c
Control
CD146+
71.44%
F-sorted
CD146+
94.5%
d
Control
CD146+
48.73%
M-sorted
CD146+
71.41%
CD 146 PE-A
